# Required peer-cooperative learning improves retention of STEM majors

**DOI:** 10.1186/s40594-017-0082-3

**Published:** 2017-09-22

**Authors:** Matthew Salomone, Thomas Kling

**Affiliations:** Bridgewater State University, Bridgewater, MA USA

**Keywords:** STEM retention, Peer-cooperative learning, Gateway courses

## Abstract

**Background:**

Peer-cooperative learning has been shown in the literature to improve student success in gateway science and mathematics courses. Such studies typically demonstrate the impact of students’ attending peer-led learning sessions on their learning or grades in an individual course. In this article, we examine the effects of introducing a required, comprehensive peer-cooperative learning system across five departments simultaneously at a master’s public university, looking not only at students’ success in supported classes, but also their retention within STEM fields two years hence. Combining institutional demographic data with students’ course grades and retention rates, we compare outcomes between 456 students who took their major’s introductory course in the two years prior to implementation of the program, and 552 students who did so after implementation.

**Results:**

While these two student groups did not significantly differ in either their demographic profile or their SAT scores, the post-implementation group earned significantly higher grades in their introductory courses in each major, due largely to an erasure of the mediating effect of SAT scores on course grades. Further, this increase in introductory course grades was also associated with an increase in the two-year retention rate of students in STEM majors.

**Conclusions:**

This finding is significant as it suggests that implementing comprehensive educational reform using required peer-led cooperative learning may have the proximate effect of mitigating differences in academic preparation (as measured by SAT scores) for students in introductory STEM courses. Furthermore, this increase in success leads to increased retention rates in STEM, expanding the pipeline of students retained in such fields.

## Background

Increasing the STEM pipeline remains an important issue and requires significant efforts to expand access and success in STEM to traditionally underserved student groups, including first-generation college students, low-income students, and students of color (National Science and Technology Council [NSTC], 2013; President’s Council of Advisors on Science and Technology [PCAST], 2012; National Center of Science and Engineering Statistics [NCSES], 2015). Unfortunately, the majority of traditionally underserved students attend under-resourced institutions whose overall graduation rates are below national averages (Association of American Colleges and Universities [AAC&U], 2015; Witham, Malcom-Piqueux, Dowd, & Bensimon, 2015; National Center for Education Statistics [NCES], 2014).

This study reports on the impact of implementing a required, institution-wide peer-cooperative learning program in science and mathematics fields on STEM retention at Bridgewater State University (BSU). BSU is a public, comprehensive four-year institution that serves large numbers of first generation college students (46.3%), low income students eligible for federal Pell grant assistance (35.4%), and underrepresented students of color (12.0%). Using a National Science Foundation’s STEM Talent Expansion Program (“STEP”) grant entitled STudent Retention Enhancement Across Mathematics and Science (STREAMS) (NSF-DUE 0969109), Bridgewater State University implemented a comprehensive approach to increasing STEM retention across five departments using a common playbook of pedagogical and co-curricular interventions, effecting a culture change in an entire college at once.

This study is significant in two ways. First, it examines a system-wide approach to using peer-cooperative learning as opposed to peer-cooperative learning in a single course or department. Second, our study reports on the level of success that can be achieved in the context of a public institution that enrolls large numbers of traditionally underserved students in STEM fields. If institution-wide, relatively low cost models such as the one reported here can be replicated across a range of universities serving large numbers of disadvantaged students, this would help to alleviate the short-fall of technically trained workers foreseen in the United States.

### Background: grant’s place in a categorization of peer cooperative learning models

Because students’ performance in gateway STEM courses has been shown to be a leading factor in STEM retention (Maton, Hrabowski III, & Schmitt, 2000; Becvar, Saupe, Noveron, & Narayan, 2008; Haak, HilleRisLambers, Pitre, & Freeman, 2011), STREAMS sought to increase STEM retention by improving student performance in gateway courses. STREAMS’s approach emphasized inquiry-based, small-group, peer-cooperative learning (Graham, Frederick, Byars-Winston, Hunter, & Handelsman, 2013; Summers & Hrabrowski, 2006; Dweck, 1986; Light & Micari, 2013; Deslauriers, Schelew, & Wieman, 2011; Froyd, 2007; Arendale, 2005), making this learning a required component of all students’ experience in gateway courses which posed the “highest risk” of attrition. In this way, STREAMS programs were similar to the Peer-Led Team Learning Model, or PLTL (Tien, Roth & Kampmeier 2002).

**Fig. 1:**
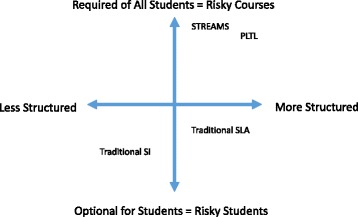
Conceptual representation of different forms of peer-cooperative learning programs, based on two dimensions: how structured the meeting time for small group is and how students enter the program. Traditional Supplemental Instruction (SI), traditional Structured Learning Assistance (SLA), Peer-Led Team Learning (PLTL) and the aggregate of the STREAMS programs at BSU are mapped

Peer-cooperative learning programs include a range of implementations that can be understood by mapping their structure and relative emphasis along two axes as in Fig. 1. Along one axis is the level of coordination and structure inherent in the peer-led sessions, and their integration with the overall instruction in the course. For example, the traditional supplemental instruction (SI; Blanc, DeBuhr & Martin 1983) and structured learning assistance (SLA; Doyle & Hooper 1997) models are differentiated by the extent to which peer-led group sessions are designed and offered independently of instructors, as in SI, or developed in coordination with, and targeted to, students on a by-section or by-instructor basis, as in SLA.

A second dimension along which one can map peer-cooperative learning programs is the extent to which the program is required of students. In both individual tutoring programs and traditional SI and SLA, students “opt-in” to the program by choosing to attend week-to-week. Alternately, only students who are at risk based on pre-identified factors are encouraged or required to attend. In other programs, students are encouraged to go to SI or SLA *after* they have done poorly on an early exam. All of these forms of entry into peer-cooperative learning are based on the concept of providing resources to support “risky students.” These forms are popular in part because they are economical; only enough resources need to be provided for students who take the effort to go or for students who are determined to be already or likely to perform poorly.

The implementation of the STREAMS program was intentionally designed to target “risky courses” and require all students enrolled in the course to attend. Based on historical data showing courses with poor performance, every section of specified courses had learning assistance attached, and students were required to attend, generally through co-registration in required cognates. Therefore, STREAMS’s version of peer cooperative learning falls on the upper half of the vertical axis in Fig. 1, as does traditional PLTL.

The courses supported at Bridgewater State University were the required introductory course for majors in biology, chemistry, computer science, mathematics (STEM-focused calculus), and physics. At the initial implementation, lackluster learning in these courses was identified as a primary barrier to student retention indicated by their D/F/W rates (percentages of students earning a D, F, or withdrawing) of 30–40%.

This “pervasive” nature of a set of inter-linked supported courses across multiple disciplines introduces a new possible axis to Fig. 1. In most implementations discussed in the literature, required peer-cooperative learning programs have been implemented only in one course or one department. So in addition to not targeting reforms to selected students in particular courses, STREAMS’s emphasis was that *every* student in *every* gateway course would participate in a peer-cooperative learning program. This “universal design” approach (Burgstahler & Cory, 2008) aimed to benefit struggling students especially by supporting all students generally, while creating an overarching network of support in both a major’s introductory and cognate courses.

Overall, Bridgewater State University grant activities and approaches were particularly inspired and aligned with PLTL and Process Oriented Guided Inquiry Learning or POGIL (Chan & Bauer, 2015; Moog, 2014). POGIL approaches were introduced through a series of discussions and professional development events including a day-long workshop with a POGIL expert. Faculty attending POGIL discussions implemented new approaches in both lecture and peer led-sessions, with particularly strong implementations in physics, mathematics and chemistry sections.

While the underlying pedagogical framework of the program was common across all five departments, each department implemented peer-cooperative learning in a fashion that best suited the strengths of its faculty and the needs of its majors (Kling & Salomone, 2015). However, departments did not vary the learning outcomes, expectations, or rigor of their course content when introducing peer-cooperative learning.

The multidisciplinary, yet differentiated, nature of STREAMS’s program is to be contrasted with peer-cooperative learning programs elsewhere in the literature, which are typically implemented in individual departments. Though the simultaneous implementation of this program across multiple STEM departments was shown to benefit majors taking required cognate courses in other fields, such as a biology major taking introductory chemistry, the current study reflects an attempt to understand student success and STEM retention when removing possible barriers to success in the gateway course to a major’s own field.

### Background: features of departmental models

The grant team helped each department to identify learning objectives currently unmet in the gateway course and develop reforms that the department could support and sustain over time. While the models adopted by each of the five supported departments varied in philosophy, structure, and scope, certain common themes helped shape a cohesive unit across departments. The primary themes were an increase in small-group work, inquiry-based learning, and some form of peer-cooperative learning, of which David Arendale’s (Arendale 2005) extensive bibliography lists several exemplar models. We were particularly interested in promoting a guided approach to inquiry, with activities directly tied to perceived departmental deficits in learning outcomes (Sadeh & Zion, 2009).

The process of working with departments to create lasting institutional change and the management of the program is described by Kling & Salomone (2015), and the models adopted by various departments are explained below. Grant leads worked to minimize potential pre/post confounding factors, such as grading bias, or changes in the components that go into course grades. We note that this long-term study could be affected by factors such as student preparation, STEM motivation or other factors outside faculty control.

#### Biology

“Biology for Life,” a weekly two-hour co-requisite course for General Biology I, was required of all students. Biology for Life utilized a case study-based curriculum and focused on study skills. Peer leaders led two course meetings per week with eight students in each. In addition to biology majors, this course was taken as a cognate by chemistry majors in a biochemistry track. Biology for Life was a one-credit, pass-fail, stand-alone course, participation in which was not included in the grade of General Biology I. There were two professors who taught General Biology I in the two years prior to implementation and the three years under study in this paper. These professors worked very closely to give similar exams throughout the study period. The exam structure did not change with a high level of correlation year to year in exam questions, nor were there any changes in the laboratory sections or syllabus pertinent to how students would be graded in the course.

#### Chemistry

Redesigned pre-labs in General Chemistry I and II replaced discussion of laboratory procedure with peer-led, inquiry-based problem-solving activities that anticipated the chemistry content of the successive lab. Peer leaders facilitated two problem sessions per week with 16 students in each. In addition to chemistry majors, this course was taken as a cognate by biology and physics majors. Participation in problem sessions did not formally add time to the student work-load, as this time had been previously scheduled but possibly under-utilized. Problem solving activities provided students with better preparation for labs and exams, but participation in those activities was not included in class grades except that students were required to attend the sessions as part of lab, and not attending would result in a failing grade in the lab. A similar set of five faculty taught sections of General Chemistry I and II during the entire study period, but faculty teaching General Chemistry I and II typically gave their own exams and typically showed independence on their choice and emphasis on topics. However, overall, there were no major changes to exams.

#### Computer science

“Introduction to Computer Science Peer Assistance” was introduced as a co-requisite for all students taking Computer Science I. Initially, this course provided peer-assisted laboratory time to work on pre-existing course projects, but over time, it developed a more conceptual, inquiry-based curriculum. Peer leaders lead three weekly 50-min meetings of eight students each. In addition to computer science majors, this course was taken as a cognate by roughly one-third of mathematics majors. This co-requisite course was a pass-fail, stand-alone course not included in the main course grade. During the sessions, peer leaders assisted students in working on projects but were directed not to provide direct solutions. Instead, over time, peer leaders developed a curriculum of similar / simpler projects that would assist students in completing main projects. Computer Science I was taught by a wide range of computer science faculty – with three common members each year – who worked with new faculty to align their courses with department and ABET accreditation-defined goals. Because the department maintained ABET accreditation, there were no major changes to the assignments or exams on which students were assessed.

#### Mathematics

“Problem Solving in Math” was a co-requisite for all students enrolled in first-semester STEM-focused calculus. A sequence of inquiry-based activities, including writing-to-learn exercises, provided deeper conceptual understanding of key material. Each peer leader led three weekly 50-min meetings of eight students each. In addition to mathematics majors, this course was taken as a cognate by all computer science and physics majors, most chemistry majors, and selected biology majors. Problem Solving in Math was a stand-alone, pass-fail course in which participation did not directly impact class grades. The introduction of this cognate did add one extra hour to student time on task, and the curriculum for the class focused heavily on inquiry-based applications of the material to assist in understanding. A team of five to six faculty taught STEM-focused calculus during the study period, and during this period there was a developing consensus on the topics, and the expectations on students was *raised* during this time period in general making the course slightly more difficult. Other than a consolidation of standards, there were no perceptible changes to the class syllabi or exams.

#### Physics

Using a “Studio Physics” model, previously-distinct lecture and laboratory modalities were combined into a single approach. In two three-hour studio class meetings per week, instruction alternated between mini-lectures, group-based inquiry activities, and laboratory-style experiments (Becvar et al. 2008). Peer leaders attended the studio classroom to assist students when working in groups on problems or labs. In addition to physics majors, this course was taken as a cognate by most chemistry majors and some computer science and mathematics majors. Students were encouraged to attend some extra time with the peer leader in the form of assignments that could be completed in small groups (outside of class) or on their own that counted towards exams by about 5 points. Since exams consisted of 70% of the course grade, this may have raised class grades by less than half a letter grade independent of any increase in learning. Two faculty taught the calculus based physics sections for the entire period of study and while there was a significant revision to the course structure, the topics and level of exams did not change.

### Infrastructure and implementation

To implement peer-cooperative learning across five departments for all students enrolled in introductory classes required a significant, but not unreasonable, development of infrastructure to support the program. STREAMS funded three weeks of summer salary for one co-I annually to oversee and train peer leaders, and the grant lead expended a significant fraction of his time during grant years 1 and 2 to working with faculty in developing structures to improve student learning.

At full implementation, approximately 25 to 30 students were employed each semester for an average of 9 h per week to provide learning assistance to a head-count of 1100 enrolled students in supported sections. These students were paid roughly $10 to $11 (US) per hour, for a total budget of nearly $60,000 (US) annually, leading to a per-enrolled student cost of about $50. After the conclusion of STREAMS, BSU has continued to fund this program, as it has been seen to be essentially cost-neutral to the university. This is because the peer-cooperative learning has led to an increase in overall student retention (which generates revenue).

BSU’s training regimen consisted of several group meetings per year that focused on small group strategies, general learning theory, and familiarizing peer leaders with institutional resources (outside the program) and when / how to refer students to those resources. Each department / faculty member was expected to meet about once per week with peer leaders to discuss learning strategies, conceptual pitfalls, and general class goals relevant to the individual field.

A non-trivial component to setting-up and maintaining the peer-cooperative learning assistance at Bridgewater consisted of developing strong relations with BSU’s Office of Institutional Research (IR) and developing skills at analysis of Institutional data. Approximately 20 hours per year of IR staff time and two weeks per year of grant lead time were dedicated to annually reviewing student success data, creating and giving presentations across campus. Faculty within the departments supported by STREAMS (but not involved in teaching the classes) and administrators benefitted from regular access to data about the success of the program. This work helped to solidify overall university support for the program and led to the program’s continued support within the departments and funding from the Institution after the conclusion of the grant funding.

### Research aims

STREAMS sought to examine whether a comprehensive, mandatory, and simultaneous approach to improving student performance in inter-linked gateway courses could lead to lasting increases in STEM retention at a university with a large percentage of traditionally underserved students. This fills a gap in the research literature by examining success across multiple departments and looking at long-term retention. Where retention rates were increased, we wished to understand the mechanism by which the increase occurred.

The overall research questions posed by STREAMS were as follows:

1. Can a systemic approach to improving student learning in gateway science and mathematics courses using a common form of peer-cooperative learning impact student grades for all populations of students?
2. Does an increase in gateway science and mathematics course success lead to long-term retention in STEM fields, and if so, by what mechanism?

The goal of STREAMS was to simultaneously change *entire department* implementations of introductory courses and to sustain that change over a long period of time (over five years in this study). We note that this could introduce a number of confounding factors – including changes in student preparation, instructor style and quality, or other factors where goals of classes “drift,” topics of emphasis change, or assessment methods of student learning vary. These possible confounding factors may have increased individual course grades and had a temporary bump in retention. For this reason, to check whether the intervention (given in the first, introductory courses) influenced long term retention, we examine two-year STEM retention. Over a two year time period, any confounding factors that led to temporarily higher grades without increases in foundational knowledge would wash away and students would not be retained.

## Methods

### Sampling

Under STREAMS, the entire gateway course in each department became supported simultaneously with peer cooperative learning. This meant that simultaneous comparison groups were not constructed, and the study was quasi-experimental. Instead, the performance and retention of students supported by the STREAMS is compared with the performance and retention of students prior to the grant. Data collection was approved by BSU’s Institutional Review Board and is available in de-identified form.^1^

Table 1 shows the number of students enrolled in each gateway course and the semester assessed as before and after implementation. All semesters when the course was offered are being assessed; the gaps in semesters assessed imply that the course was not offered at that time.

**Table 1:** Total numbers of students and numbers of majors (e.g. chemistry majors taking gateway chemistry) before and after implementing peer cooperative learning. Only 15-week fall and spring academic semesters are included

	Before Implementation			After Implementation		
Gateway Course	N		Semesters Assessed	N		Semesters Assessed
	Majors	Total		Majors	Total	
Biology	131	196	F08, F09	274	386	F10, F11, F12
Chemistry	49	267	F09, F10	42	363	F11, Sp12, F12
Comp. Science	112	365	F09–F11	74	257	Sp12, F12, Sp13
Mathematics	146	546	F09–Sp11	135	379	F11–Sp13
Physics	18	162	F09–Sp11	27	115	F11–Sp13
*Total*	*456*	*1536*		*552*	*1500*	

In this study looking at retention within a STEM major, students were included who were declared STEM majors taking their program’s gateway course for the first time (for instance, a declared chemistry major taking introductory chemistry), and divided into the group of these students who took the course during the two years prior to implementation of peer-cooperative learning (*N* = 456) and the two years after (*N* = 552).

Demographic data, SAT scores, and two years’ worth of academic records were collected for all students. Over the time period studied, enrollment in these courses increased due to university-wide growth in student headcount. However, there were no changes in admissions policies and no significant differences in academic preparation or demographic profile in the pre- and post-implementation groups as is shown in Table 2. As students are able to elect not to complete certain demographic data questions, there are some students for whom we do not know income status or ethnicity. Where analysis relied on those markers, these students were excluded from the sample.

**Table 2:** SAT scores and demographic factors for students pre- and post-implementation. *N* refers to the number of students for whom data on each factor was available, and the percentage listed is that fraction of the sample for the category listed. No pre- to post-implementation differences were statistically significant

	Pre-implementation			Post-implementation		
All Students	*N*	Mean	S.D.	*N*	Mean	S.D.
SAT-Math	383	526	113	466	520	116
SAT-Verbal	376	498	89	461	498	105
Gender (Female %)	456	50%		552	54%	
Ethnicity (Nonwhite %)	419	19%		427	21%	
First-generation (Yes)	401	50%		530	47%	
Low-income (Yes)	237	37%		293	37%	

Of particular interest to us is tracking cohorts of majors as they progress through their studies. We see virtually no differences between our cohorts in participation rates of women, low-income students, first-generation students, and under-represented minorities overall in our student population. Overall, 55.5% of the majors in the gateway courses before the intervention and 54.7% of the students after fall into one or more of the traditionally underserved categories of low income, first generation, or underrepresented students of color. Women, first generation students and low-income students are sizeable proportions of the student population and, as is typical of our institutional classification, are over-represented as a proportion of the students enrolled (as compared with more selective institutions).

One might also posit that incoming students after the implementation of STREAMS were significantly stronger, and therefore more likely to be retained. There were no changes in the university admission policies during the time period of the grant and no significant changes in the course pre-requisites for the gateway courses. The College of Science and Mathematics does not have different admissions policies from the university overall. We show in Table 3 that no significant difference is present in the incoming student SAT scores, which are the best proxy available to us for student preparation. Since SAT scores, particularly SAT-Math scores, are often correlated with gateway course success, we will retain SAT-Math as an independent statistical control whenever course success is an outcome. Still, the data in Tables 2 and 3 indicate that, both in the aggregate and in each individual major, there was not a statistically significant “background” difference in the demographics or academic preparation of students between pre- and post-implementation that might otherwise have contributed to a differential in gateway course success or retention in a STEM among any subgroup of majors studied.

**Table 3:** The average incoming SAT scores of majors in the gateway course listed, with the standard deviation of the sample indicated. Overall, no meaningful change is present in the SAT scores of incoming students in the gateway courses

Gateway Course	SAT Math (before)	SAT Math (after)	SAT Verbal (before)	SAT Verbal (after)	SAT Total (before)	SAT Total (after)
Biology	519 ± 75	510 ± 72	505 ± 73	505 ± 82	1028 ± 138	1017 ± 137
Chemistry	541 ± 70	552 ± 68	500 ± 93	519 ± 79	1040 ± 148	1072 ± 126
Comp. Sci.	541 ± 75	550 ± 83	506 ± 79	529 ± 83	1046 ± 139	1079 ± 149
Mathematics	552 ± 86	561 ± 70	493 ± 73	482 ± 78	1048 ± 128	1043 ± 126
Physics	575 ± 48	594 ± 85	545 ± 92	564 ± 78	1121 ± 133	1158 ± 142
Overall	539 ± 79	535 ± 77	502 ± 77	506 ± 83	1043 ± 136	1041 ± 139

### Measurements

Three outcomes were studied for each student: their gateway course grade (on a four-point scale with A = 4.0 and F = 0.0), whether their grade represents a “successful” outcome (a dichotomous measure defined as a grade of B-minus or higher), and whether the student was still an active STEM major two years later.

The two-year time period is chosen as a proxy for matriculation into junior-level coursework, a key indicator of success and future degree completion. We define a major taking a gateway course as having been retained in STEM if, two years (i.e. four regular semesters) later, they remained a STEM major, continuing to take STEM classes. This definition disregards changes of major *within STEM* as not relevant to the analysis.

### Limitations of the current study

The primary goal of STREAMS was institutional change, and as a result, the current study has several limitations. The data collected will allow us to test the fundamental research questions of this paper, and we will be able to examine the impact of class success on retention through our statistical modeling. However, we did not seek to understand particular aspects *within* the curricular change of adding peer-cooperative learning that might have been more important to increasing STEM retention. For example, students were not randomized to some peer cooperative learning groups that increased time on task or an equal time (relative to the prior teaching strategies). Therefore, we cannot determine whether our resulting increased grades or retention are due to increased time with the students or some other factor. Because we supported all the sections of all the instructors of introductory courses, the natural variation in teaching across instructors and sections over multiple years makes it difficult to compare year-to-year exams.

Given these limitations in determining the details of *why* the program worked, we will focus on the long-term, longitudinal outcome – did the inclusion of peer-cooperative learning increase the rates at which students were retained multiple years later. In part, our examination of 2-year retention rates – retention into the “junior” year – is designed to eliminate noise that might come from variations in success in individual classes at the outset of study.

### Analytic approach

Because we wish to propose a mechanism by which rates of gateway course success and STEM retention were affected by students’ participation in required peer-cooperative learning, bivariate correlation will be used to identify significant correlations between these rates and a variety of student-level factors that include both demographic variables and academic variables (the latter in the form of SAT exam scores).

Where significant correlations exist, a binary logistic regression will help to quantify the effect sizes of these variables on success and retention, and compare these effects in the pre- and post-implementation groups. Logistic regression is a well-known technique which can determine how strongly variables influence dichotomous outcomes such as retention or success (Cabrera 1994, Peng, Lee & Ingersoll 2002). We coded gateway course “success” as the attainment of a course grade of B-minus (roughly 80% of available course credit earned) or greater, which augurs well for a student’s successful completion of the subsequent semester in the introductory sequence and their preparation for more specialized coursework in the following years of the major.

## Results

### Course grades, success & retention

Course grades, course success rates and STEM retention rates increased for STEM majors overall, and in four out of five supported departments, after the STREAMS program was implemented (Table 4). Because implementation began at different times across departments, the number of cohorts used the net increase of majors column varies.

**Table 4:** The GPA and course success rates (defined as a grade of B- or better) for majors in the supported gateway course. Also presented are two year retention rates within STEM, the percentage point increase in the retention rate, and the number additional number of majors retained per year. Significant differences are marked with * (*p* < 0.05) and ** (*p* < 0.01)

	Course Grades				Two-Year STEM Retention			
Department	GPA		Success Rate		Retention Rates		Net Increase	
(N pre / N post)	Before	After	Before	After	Before	After	Rate	Majors
Biol. (131/274)	2.12	2.52**	42.0%	56.6%**	45.0%	56.6%*	+11.6%	+11
Chem. (49/42)	2.24	2.99**	55.1%	71.4%	46.9%	69.0%*	+22.1%	+5
C. Sci. (112/74)	2.13	2.23	52.7%	54.2%	35.7%	45.9%	+10.2%	+5
Math (146/135)	2.33	2.80**	53.4%	65.9%*	55.5%	63.7%	+8.2%	+6
Physics (18/27)	2.11	2.42	55.6%	48.1%	77.8%	70.4%	−7.4%	−1
*Total (456/552)*	*2.20*	2.58**	*50.2%*	59.3%**	*48.0%*	59.0%**	*+11.0%*	*+25*

The aggregate increase in course success rate and STEM retention rate across the college was statistically significant, driven by significant increases in each of the subgroups of students taking introductory biology, chemistry, and calculus. The increases in these courses correlated with an increase in overall retention of students in STEM majors by 25 students annually.

Interestingly, while retention of physics majors declined, non-physics STEM majors taking physics as a cognate improved substantially: the AB success rate for all STEM majors in General Physics I increased from 32% (*N* = 162) to 47% (*N* = 115). As physics was the department with the smallest number of majors, the departmental decline, which was not statistically significant and consisted of a swing of roughly one to two students performing below expectations, did not impact overall increases. Physics majors also had the highest retention rates to begin with, so that there may have been a ceiling effect to the intervention.

### Interplay between student preparation, demographics and success

Bivariate Pearson correlation coefficients were examined to quantify the strength of the relationship of student preparation and demographic variables to one another, as well as to students’ gateway course performance and STEM retention. The correlation coefficients are shown in Table 5. With respect to independent factors, there were significant bivariate correlations between students’ ethnicity, low-income status, and first-generation status, with nonwhite students disproportionately likely to have first-generation and low-income status. These demographic factors, along with gender, were each negatively correlated with SAT-Math and SAT-Verbal scores both pre- and post-implementation, although this association was less significant among first-generation students than among female, nonwhite, and low-income students.

**Table 5:** Pearson pairwise correlations between demographic factors, SAT scores, and outcome variables pre- and post-implementation. Significant correlations are marked * (*p* < 0.05) and † (*p* < 0.01)

	Demographic Factors			SAT Scores			Gateway Course		
	Ethnic.	1st Gen.	Low-in.	Math	Verbal	Total	Grade	Success	2Y Ret.
Pre-implementation									
Gender (1 = Female)	.01	−.03	.07	−.15^†^	−.11^*^	−.18^†^	−.03	−.02	.04
Ethnicity (1 = Nonwhite)		.14^†^	.18^†^	−.22^†^	−.23^†^	−.32^†^	−.10^*^	−.11^*^	−.11^*^
First-generation			.27^†^	−.03	−.06	−.09	.07	.05	.12^*^
Low-income status				−.11	−.22^†^	−.27^*^	.02	−.05	.07
SAT-Math score					.70^†^	.57^†^	.17^†^	.17^†^	.15^†^
SAT-Verbal score						.83^†^	.20^†^	.21^†^	.07
SAT total score							.28^†^	.31^†^	.14^†^
Gateway course grade (4.0 = A)								.86^†^	.49^†^
Gateway success (1 = B– or higher)									.41^†^
2-yr. STEM retention (1 = retained)									
Post-implementation									
Gender (1 = Female)	.08	.08	.08	−.16^†^	−.11^*^	−.22^†^	.06	.05	.03
Ethnicity (1 = Nonwhite)		.19^†^	.34^†^	−.27^†^	−.30^†^	−.37^†^	−.17^†^	−.17^†^	−.13^†^
First-generation			.27^†^	−.11^*^	−.11^*^	−.18^†^	−.01	−.02	−.03
Low-income status				−.07	−.22^†^	−.22^†^	−.11	−.11	−.10
SAT-Math score					.73^†^	.58^†^	.17^†^	.15^†^	.07
SAT-Verbal score						.73^†^	.15^†^	.15^†^	.08
SAT total score							.34^†^	.33^†^	.22^†^
Gateway course grade (4.0 = A)								.84^†^	.51^†^
Gateway success (1 = B– or higher)									.44^†^
2-yr. STEM retention (1 = retained)									

There was a significant amount of mutual correlation between the demographic variables in the data (gender, ethnicity, first-generation status, and low-income status), due to the fact that students of color in the study were more likely to have first-generation and low-income status. To mitigate this effect, a principal component analysis was used (Table 6) to combine these four dichotomous variables into a single factor score hereafter called “Demographics.” This score may be interpreted as a measure of intersectionality between the three demographic factors: the score increases for each of the categories (female, nonwhite, first-generation, low-income) into which a student falls.

**Table 6:** Principal component analysis on the four demographic factors in the study. The principal component among these variables explains 37.7% of total variance

	Pearson Correlation Coefficient			Principal
	Ethnicity	First-Gen.	Low-Income	Component
Gender (1 = female)	0.052	0.046	0.082	0.249
Ethnicity (1 = nonwhite)		0.186	0.266	0.663
First-Generation (1 = yes)			0.274	0.669
Low-Income (1 = yes)				0.748

With respect to the outcome variables, students’ gateway course grade and success rate were most strongly correlated (on a bivariate basis) with SAT scores, both math and verbal subtests individually, as well as their sum total. Ethnicity was also correlated with gateway course performance both before and after implementation; however, we will see that this is an indirect effect mediated by SAT scores. Finally, retention in STEM two years after taking the gateway course was most strongly correlated with gateway course performance, reflecting existing literature on retention. As such, retention was also correlated (likely indirectly) with ethnicity and SAT scores.

### Linking course grades and success to retention

Multivariate logistic regression models were created for two-year STEM retention against Demographics, SAT scores, and gateway course grades in both pre- and post-implementation cases. In both cases, the correlation is nontrivial (Nagelkerke’s *r* ^2^ ≈ 0.219 pre, 0.286 post), and among the predictor variables only the gateway course grade is significantly correlated to STEM retention. These models are shown in Table 7. The correlation between gateway course grade and retention in both pre-implementation (Odds Ratio 1.98–8.16, *p* < 0.001) and post-implementation (Odds Ratio 4.27–14.8, *p* < 0.001) is significant in both models. In other words, a one-letter increase in gateway course grade was correlated with a twofold or greater increase in the odds of a student being retained in STEM two years later. There is not sufficient evidence to conclude that this relationship changed from pre- to post-implementation. We infer that there were no structural changes during this period that made gateway course performance significantly more or less predictive of STEM retention.

**Table 7:** Logistic regression coefficients with dependent variable STEM retention after two years. Gateway A/B grades are the only significant predictor of STEM retention, and its effect size does not significantly differ pre- to post-implementation

Factor	Coefficient B	Significance *p*	Odds Ratio	95% CI
Pre-implementation Nagelkerke *r* ^2^ = 0.219				
Demographics	0.306	0.132	1.36	0.91–2.02
SAT-Math (std.)	0.702	0.054	2.02	0.99–4.12
SAT-Verbal (std.)	−0.399	0.143	0.671	0.39–1.14
*Gateway A/B grade*	*1.390*	*<0.001*	*4.02*	*1.98–8.16*
(Constant)	−0.894	0.001		
Post-implementation Nagelkerke *r* ^2^ = 0.286				
Demographics	−0.218	0.163	0.804	0.59–1.09
SAT-Math (std.)	0.157	0.526	1.17	0.72–1.90
SAT-Verbal (std.)	−0.015	0.943	0.986	0.66–1.47
*Gateway A/B grade*	*2.071*	*<0.001*	*7.935*	*4.27–14.8*
(Constant)	−0.0962	<0.001		

Hence, the increase in STEM retention was a result of increased gateway course performance. But what explains this increase in performance? Table 8 displays the results of logistic regressions of gateway course success against demographics and SAT scores. Here, there is a dramatic difference in the correlative strength of the model between the pre-implementation and post-implementation groups. Prior to implementation, students’ SAT-Math scores were a highly significant correlate of course success (Odds Ratio 1.78–7.69, *p* < 0.001), such that an increase of one standard deviation correlated with roughly a nearly fourfold increase in the likelihood of a student earning a B-minus or better in the course. That is, students’ academic preparation (at least with regard to mathematical skill) was a significant predictor of whether they would succeed in their gateway course.

**Table 8:** Logistic regression coefficients with dependent variable gateway course success (B– or greater). Pre-implementation, demographic factor and SAT-Math scores were significantly correlated with gateway course success. Post-implementation, SAT-Math scores are no longer significant predictors of gateway course success

Factor	Coefficient B	Significance p	Odds Ratio	95% CI
Pre-implementation Nagelkerke *r* ^2^ = 0.165				
*Demographics*	*0.442*	*0.028*	*1.56*	*1.05–2.31*
*SAT-Math (std.)*	*1.309*	*<0.001*	*3.702*	*1.78–7.69*
SAT-Verbal (std.)	−0.088	0.745	0.915	0.54–1.56
(Constant)	−0.256	0.183		
Post-implementation Nagelkerke r^2^ = 0.029				
Demographics	−0.111	0.437	0.895	0.68–1.18
SAT-Math (std.)	0.209	0.372	1.232	0.78–1.95
SAT-Verbal (std.)	0.090	0.634	1.095	0.75–1.59
(Constant)	0.611	<0.001		

Post-implementation, however, the correlation of this multivariate model is negligible (*r* ^2^ ≈ 0.029) and *none* of the predictors achieve statistical significance. Among students who participated in the peer-cooperative learning program, there is insufficient evidence to conclude that either demographic factors *or* their SAT scores bore upon their likelihood to succeed in their gateway course. This suggests that the significant mediating effect that SAT-Math scores had on gateway course success (and consequently on STEM retention) prior to implementation is no longer significant post-implementation.

More specifically, the pre-implementation model indicated that both students’ demographics and their SAT-Math scores were independently and significantly correlated with their success in gateway courses. That neither were statistically significant independent predictors of success post-implementation is likely an artifact of multicollinearity among these two factors. It does not mean, for instance, that students across the spectrum of SAT-Math scores were equally successful in their gateway course, or retained in STEM in equal proportions, post-implementation. Table 9 shows the mean gateway course grade, gateway course success rate, and STEM retention rate for students in the lowest and highest quartiles of SAT-Math scores. The rising tide has indeed “lifted all boats”: both of these quartiles saw an average increase of one-half letter grade and approximately an 11 percentage-point increase in gateway course success rate and STEM retention rate. However, there were significant gaps between the quartiles in all three outcomes both pre- and post-implementation suggesting that the independent effects of demographics and SAT-Math scores pre-implementation may have become a joint effect post-implementation.

**Table 9:** Gateway course outcomes and STEM retention rates for students in the lowest and highest quartile of SAT-Math scores, pre- and post-implementation. In both groups, quartile 1 included SAT-Math scores of 0–480 and quartile 4 included scores of 590–800. *Pre- to post-implementation *t*-statistic indicated significant differences, *p* < 0.05

		Gateway Course Grade			2-Year STEM
	N	Mean	S.E.	Success	Retention
Pre-Implementation Total	456	2.20	0.07	50%	48%
*SAT-Math quartile 1*	102	1.69	0.12	28%	32%
*SAT-Math quartile 4*	102	2.65	0.15	67%	57%
Post-Implementation Total	552	2.58	0.05	59%	59%
*SAT-Math quartile 1*	143	*2.11	0.09	39%	44%
*SAT-Math quartile 4*	143	*3.06	0.11	*79%	*71%

### Summary of significant results

Taken together, the results of this study indicate that (Committee of STEM Education, National Science and Technology Council, 2013) the demographics and academic backgrounds of the STEM majors supported by GRANT’s intervention did not significantly differ from that of STEM majors prior to STREAMS; (President’s Council of Advisors on Science and Technology, 2012) as suspected, poor performance in gateway courses was, and remains, a significant predictor of attrition from STEM; (National Center for Science and Engineering Statistics, 2015) the introduction of a system of peer-cooperative learning did improve student retention; and (Association of American Colleges and Universities, 2015) academic background, specifically a student’s SAT-Math score, a significant correlate of STEM retention in the pre-implementation group, was no longer independently correlated to STEM retention in the post-implementation group.

## Discussion

### Discussion: holistic approach

The findings at Bridgewater State University support a holistic approach to STEM retention through making systemic changes to gateway courses (Malcom & Feders, 2016). We note that the STREAMS approach differs from much of the literature in two significant ways. First, we required all students to participate in the interventions instead of targeting subsets of instructors or students. Second, we simultaneously created a system of similar, complementary supports across multiple departments, so that students were supported in their required major and cognate courses.

The holistic approach that was successful at BSU was accomplished by both insisting that all departments participate in a peer-cooperative learning program and allowing departments freedom within that framework to develop a model that worked within their local situations. Different faculty, and different departmental faculty cultures, were accommodated by allowing the department faculty who regularly taught the supported courses to create their version of peer-cooperative learning. These faculty then “sold” the program to their departmental colleagues. The STREAMS team worked across departments to share strategies that were seen by the leadership team as strong, and by doing so, nudged departments towards more commonality, particularly as time went on.

By working with departments to either create required cognate courses or transform existing class time, BSU has been able to create a system that is required of all students and not “optional,” designated for “at risk” students, or a program for students already identified as struggling – all of which are more common for typical Structured Learning Assistance or Supplemental Instruction approaches (Arendale 2005). Overall, the program was not particularly costly – the total cost per student worked out to be about $50 (U.S.) per student enrolled in the supported course.

### Discussion: factors leading to success

In our study, logistic regression models establish that there exists a causal relation between the increase in the number of students who earned high grades and the number of students who were retained in STEM fields. Among demographics, SAT scores, and gateway course grade, the only significant predictor of STEM retention into the 3rd year was the gateway course grade within both the pre-intervention cohort and the post-intervention cohort. Students earning D, F, or W grades continued in STEM into the third year 15.1% (14.3%) post- (and pre-) intervention. Meanwhile, students earning A or B grades were retained in STEM to year three 68.1% of the time before and 76.7% of the time after the introduction of STREAMS’s peer-cooperative learning, where this difference in retention is itself statistically significant. Therefore, we emphasize two important effects on STEM retention: first *more* students earned A and B grades, and a *greater percentage* of high performers were retained in STEM.

When we examine whether demographics or student preparation (SAT scores) impact success in the gateway course, we see an interesting effect. Higher SAT math scores strongly predicted gateway course success prior to STREAMS and did *not* predict gateway success after the introduction of peer-cooperative learning support.

This finding suggests that the mechanism by which this peer-cooperative learning enhanced STEM retention is by removing the mediating effect of SAT scores on students’ gateway course success. That is, peer-cooperative learning has compensated for uneven student preparation within the gateway course and helped all students — but especially students with lower SAT scores — to earn the successful grades in these courses that are correlated with retention in STEM.

Student opinion on the causes of their retention in STEM fields seems to align with the analysis presented here. In preparation for the submission of STREAMS, the BSU Office of Institutional Research conducted a survey of 114 students who began as STEM majors but changed to non-STEM fields. In this survey, 65% of respondents indicated that lack of success in introductory courses was a significant factor in changing majors. This compares to 42% who indicated a lack of mentoring was relevant, 29% who indicated concerns with total course load, and 15% who cited poor course instruction. By using the peer-cooperative learning approach, GRANT sought to improve course performance and provide more opportunities for mentoring.

At the conclusion of the period studied here, a survey was given to science and math majors who had participated in STREAMS’s activities. In this survey, 79% of students who had taken courses supported by peer cooperative learning indicated that they agreed (39/102) or strongly agreed (40/102) with the statement that peer-cooperative learning support “significantly aided me in learning science and mathematics in the intrpductory course.” Also, 74% indicated that they agreed (34/103) or strongly agreed (42/103) with the statement that peer-cooperative learning “helped me be more successful as a science or math major.”

### Conclusions

While other institutions attempting to re-create the program described in this paper might see the “up-front” work of convincing colleagues across departments to agree to a more or less common approach, we feel that that the data presented in this study indicate that a more holistic approach can lead to better STEM retention. This is particularly true if one looks at retention in STEM – possibly across STEM fields – as the main goal, as opposed to retention in a particular department.

Students in our study are supported in more than one class. They receive support in their initial gateway course – where we see that they achieve higher grades and a higher success rate (B- or better). But they also receive support in other cognate courses taken in the early years – for instance in chemistry courses for biology majors, or calculus courses for physics and computer science majors. By getting accustomed to a peer-cooperative style of learning in multiple settings, we feel that students were more quickly able to adjust to new course content and ways of thinking. Future studies of the success of students taking cognate classes supported by a required, peer-cooperative learning program would help to clarify whether the impact on retention was due more to support in the initial gateway course or in the cognates.

Nevertheless, the results of our study indicate that required peer-cooperative learning programs across departments can alleviate preparation deficits and lead to increases in retention in STEM fields into “junior year” status. Similar institutions serving a range of traditionally underserved students may benefit from cross-departmental programs of support such as the one created by GRANT.

## Footnotes

1 This data is GPL-licensed and may be downloaded at https://figshare.com/articles/Required_Peer_Cooperative_Learning_STEM_Retention_Data/4239677 .
